# Functional Ginger Extracts from Supercritical Fluid Carbon Dioxide Extraction via *In Vitro* and *In Vivo* Assays: Antioxidation, Antimicroorganism, and Mice Xenografts Models

**DOI:** 10.1155/2013/210845

**Published:** 2013-07-29

**Authors:** Chih-Chen Lee, Li-Yu Chiou, Jheng-Yang Wang, Sin-You Chou, John Chi-Wei Lan, Tsi-Shu Huang, Kuo-Chuan Huang, Hui-Min Wang

**Affiliations:** ^1^Department of Chemical Engineering, National Chung Hsing University, Taichung 402, Taiwan; ^2^Derlin Biotech Corporation, Nantou 540, Taiwan; ^3^Department of Fragrance and Cosmetic Science, Kaohsiung Medical University, Kaohsiung 807, Taiwan; ^4^Department of Biotechnology, College of Life Sciences, Kaohsiung Medical University, Kaohsiung 80708, Taiwan; ^5^Department of Chemical Engineering and Materials Science, Yuan Ze University, Taoyuan 32003, Taiwan; ^6^Section of Microbiology, Department of Pathology and Laboratory Medicine, Kaohsiung Veterans General Hospital, Kaohsiung 807, Taiwan; ^7^Department of Medical Technology, Fooyin University, Kaohsiung County 831, Taiwan; ^8^School of Medicine, National Yang-Ming University, Taipei 112, Taiwan; ^9^Department of Bioindustry Technology, Dayen University, Changhua 515, Taiwan; ^10^Graduate Institute of Natural Products, Kaohsiung Medical University, Kaohsiung 80708, Taiwan

## Abstract

Supercritical fluid carbon dioxide extraction technology was developed to gain the active components from a Taiwan native plant, *Zingiber officinale* (ginger). We studied the biological effects of ginger extracts via multiple assays and demonstrated the biofunctions in each platform. Investigations of ginger extracts indicated antioxidative properties in dose-dependant manners on radical scavenging activities, reducing powers and metal chelating powers. We found that ginger extracts processed moderate scavenging values, middle metal chelating levels, and slight ferric reducing powers. The antibacterial susceptibility of ginger extracts on *Staphylococcus aureus*, *Streptococcus sobrinus*, *S. mutans*, and *Escherichia coli* was determined with the broth microdilution method technique. The ginger extracts had operative antimicroorganism potentials against both Gram-positive and Gram-negative bacteria. We further discovered the strong inhibitions of ginger extracts on lethal carcinogenic melanoma through *in vivo* xenograft model. To sum up, the data confirmed the possible applications as medical cosmetology agents, pharmaceutical antibiotics, and food supplements.

## 1. Introduction

Antioxidative components are of importance due to the abilities to reduce free radical-mediated degradations of cells, tissues, and organisms in humans [1]. There are many diet sources to decrease oxidative stress, including legumes, cereals, fruits, various vegetables, and other herbal medicines all over the world [2]. 

With the long-term exposure in high oxidative stress, the normal cells often accompany with carcinomas and transfer into sick status. The strategy of diminishing oxidative stress is a good solution to keep human being physiologically healthy. Therefore, natural antioxidants from plant species which decrease oxidative stress from intrinsic and external sources have huge applicable biofunctions in human health care [3, 4].

A worldwide troubling public health issue emerged from hospital-acquired or nosocomial illness is caused by infections microorganisms in recent decades. Pathogenic bacteria are able to survive for extended periods on human superficial skin, mucosa, or environmental surfaces and have been implicated in infectious outbreaks within hospitals, medical facilities, and institutions in many countries [5]. With the abuse of broad-spectrum antibiotics, the most alarming characteristic of these microorganisms is their resistance to almost all commercially available antimicrobial drugs. As a result, many of these microorganisms were now classified as highly antibiotic-resistant microorganisms. Therefore, the research requirements for new anti-infection therapeutic agents have increased in natural medicinal therapies. The utilization of plant-derived constitutes as preservatives, cosmetics, and pharmaceuticals has recently attracted increased interests [6].

Human skin is normally contacted with damage stress, which is produced by external and intrinsic sources, such as ultraviolet radiation, free radicals, and reactive oxygen species. There are many studies about the ultraviolet radiation which are responsible for skin aging or tumorigenesis. Melanoma, a malignant tumor of epidermal melanocytes, is one of the most deadly skin cancers [7]. During the past several decades, the occurrences of cutaneous melanoma have increased because it has a strong propensity to metastasize and, therefore, is one of the most aggressive skin cancers. Unlike other cancers, the malignant melanoma is not easy to treat with surgery, chemotherapy, or radiotherapy [8]. Clinically, recent treatments for advanced melanoma do not significantly improve the mortalities and prolong survivals of patients due to the exceeding metastasis of melanoma [9]. Fortunately, numerous widely used Chinese medicinal herbs have been shown to exert anticancer bioactivities [10, 11]. For this reason, the development of herbal medicine has the possibility of the therapeutic effects against melanoma cells.

Traditionally, natural matrices have been obtained by means of conventional extraction with organic solvents. Nevertheless, the extraction changes in the physicochemical properties of extracts could alter their functionalities. Thus, the extraction processes should be performed at suitable and mild conditions. Supercritical carbon dioxide extraction is an advanced technology that has many advantages, including low environmental impacts due to no residue of harmful solvents, noncorrosiveness, nontoxicity, and easy separation from extracts [12, 13]. It was widely used in pharmaceutical and food industries in modern times [14].

Ginger, the powdered rhizomes of the herb* Zingiber officinale* Roscoe (Zingiberaceae, dietary ginger), is used widely as a spice throughout the world. In Chinese medicine, ginger has been used traditionally as a treatment for allergy, constipation, asthma, diabetes, nervous diseases, rheumatism, toothache, stroke, and antimicroorganism infection [15]. In addition, ginger could be as the treatment of chemotherapy-associated nausea, the suppression of platelet aggregation, the inhibition of tyrosinase, cyclooxygenase, or nitric oxide synthase and prevent lipid peroxidation which sets in a variety of biofilm by oxidation to cause injury [16–18]. The previous literature reported about inhibiting skin tumor promotion in imprinting control regions mice [19]. The present work analyzed ginger extracts to identify the antioxidative, antimicrobial, and anticancer activities.

## 2. Materials and Methods

### 2.1. Reagents and Samples

Dimethyl sulfoxide (DMSO) and Luria-Bertani broth were purchased from Sigma-Aldrich Chemical Inc. (St Louis, MO, USA). Dulbecco Modified Eagle Medium (DMEM), fetal bovine serum (FBS), penicillin, and streptomycin were purchased from Life Technologies CO., Ltd. (Gibco, Grand Island, NY, USA). The plant specimen was authenticated by Dr. Chih-Chen Lee, Derlin Biotech Corporation (Nantou, Taiwan), where voucher specimens were kept. The air-dried extracts of ginger were cultivated and obtained in the district of Nantou, Taiwan (2012). All buffers and other reagents were of the highest purity commercially available.

### 2.2. Supercritical Extraction

The supercritical carbon dioxide fluid extraction was performed on United States supercritical fluid extractor (Applied Separation Inc. Co. Ltd.; Derlin Biotech Corp. Taiwan) with the extractor volume of 5.0 L. The liquid ethanol from a cylinder was pressured to reach the supercritical state with a piston pump and cooling at −4°C in a water bath before it passed into the extraction vessel (with 5.0 L inner extraction container). The extraction pressure was adjusted by needle and micrometric valves. The extraction temperature was controlled by a thermostatic batch. The flow rate of ethanol was regulated by a rotameter. The container was placed into the extraction vessel, and the consumption volume of ethanol was recorded by a gas meter. After the temperature of the extraction vessel reached the set point, the micrometric valves were closed. Then, ginger extracts (500 g) were loaded into a 5.0 L stainless steel extraction container (9.2 cm ID) with stainless steel filters placed at both ends to prevent carryover of the particles. The pressure of extraction vessel was controlled at 5,800 psi by a needle valve and set at 2 hours of static extraction time with a timer. The solute-rich fluid departing from the extractor was expanded through micrometric valves to atmospheric pressure. The flow rate was set at 6 NL/min and the total volume of gas was measured with a gas meter. The carbon dioxide discharge was recycled to cultivate ginger extracts to reduce carbon dioxide emission to the environment.

### 2.3. Determination of DPPH Radical Scavenging Capacity

DPPH is a stable free radical with violet color (absorbed at 517 nm), and DPPH solution changes its color to light yellow when free radicals are scavenged. Various concentrations of ginger extracts (100~5000 *μ*g/mL) were added to 1 *μ*L of stable DPPH (60 *μ*M) solution and measured with a 96-well plate. When DPPH radicals react with antioxidative agents donating hydrogen, the solution color is reduced resulting in a decrease in absorbance at 517 nm (Table 1). The analyzed time interval was 5 min per point, up to 30 min by using UV-vis spectrophotometer (BioTek Co.). Vitamin C was used as a positive control. The DPPH radical scavenging activity (%) was determined as

(1) $$\begin{array}{c} \text{scavenging}\;\;\text{activity}\%=\frac{\left(A_{\text{control}}-A_{\text{samples}}\right)}{A_{\text{control}}}\times 100\%. \end{array}$$

### 2.4. Metal Chelating Activity

The ferrous ion chelating potential from ginger extracts (100~5000 *μ*g/mL) was investigated according to a previously described method [20]. Briefly, testing samples dissolved in DMSO were added to a solution of 2 mM FeCl_2_·4H_2_O (10 *μ*L). The reaction was initiated by the addition of 5 mM ferrozine (20 *μ*L), and the mixture was vigorously shaken and left standing at 25°C for 10 min. The absorbance of the mixture was read at 562 nm against a blank. EDTA was used as a positive control, and the chelating activity calculation formula was similar to (1).

### 2.5. Reducing Power Assay

The reducing powers of ginger extracts (100~5000 *μ*g/mL) were determined according to the method of [21]. In brief, testing compounds were mixed with 85 *μ*L of 67 mM phosphate buffer (pH 6.8) and 2.5 *μ*L of 20% K_3_Fe(CN)_6_. The mixture was incubated at 50°C for 20 min, and then 160 *μ*L of trichloroacetic acid (10%) was added to the mixture to centrifuge for 10 min at 3,000 ×g. The upper layer of the solution (75 *μ*L) was mixed with 2% FeCl_3_ (25 *μ*L), and the absorbance was assayed with a 96-well plate at 700 nm. A higher absorbance demonstrates a higher reductive capability. BHA was used as a positive control.

### 2.6. Microorganism Strains

The four microorganismic strains used within this study, which were purchased from American Type Culture Collection (ATCC), included *Staphylococcus aureus* (ATCC 29213), *Streptococcus mutans* (ATCC 25175), *S. sobrinus *(ATCC 33478), and *Escherichia coli* (ATCC 35218). The most common species of *staphylococcus* is *S. aureus*, which causes staphylococcus infections and is frequently found in the human respiratory tract and on the skin surfaces. The emergence of antibiotic-resistant forms of pathogenic *S. aureus* (e.g., methicillin-resistant *Staphylococcus aureus*, MRSA) is a worldwide problem in clinical medicine [22]. Another species in the *Streptococcus* species is *S. sobrinus* which is a spherically shaped anaerobic and Gram-positive bacterium. They grow in pairs or chains, and they are not motile and do not form spores. *S. mutans* is a facultatively anaerobic, Gram-positive, coccus-shaped bacterium commonly found in the human oral cavity and is a significant contributor to tooth decay. The most intensively studied prokaryotic model organism is *E. coli*, a Gram-negative, rod-shaped bacterium commonly found in the lower intestine of warm-blooded organisms. This bacterium is easily and inexpensively grown in a laboratory setting and has been studied intensively in the past half century [23].

### 2.7. Determination of Antibacterial Properties

The antimicrobial properties of the ginger extracts were investigated using previously described methods. Briefly, 10^5^ CFU/mL microbial suspensions of four bacteria, *S. aureus*, *S. mutans*, *S. sobrinus,* and *E. coli*, were incubated in each well at 37°C for 24 hours. The microbial bacteria were then harvested in normal saline and adjusted to McFarland 0.5 (1.5 × 10^8^ CFU/mL). One milliliter of each bacterial sample suspension was centrifuged at 9,000 rpm for 5 minutes and then treated with 0.3 mL of an extract to obtain a final concentration of 10% v/v. Reactions were compared at 25°C at intervals of 5, 30, 60, 180, 300, and 900 seconds, respectively. A well containing DMSO was used as the growth blank vehicle control; a well containing medium only was used as the negative control; all other wells contained the experimental groups treated with ginger extracts. The examination was considered valid if the well for the growth control group was positive and those for other groups were negative. After the specified reaction times, the bacteria were centrifuged for 2 minutes, washed once, and suspended in sterile saline water. A 10^4^-fold dilution of the bacterial suspension (100 *μ*L) was then plated on blood agar plates. After 24-hour incubation period time, the bactericidal effects of the extracts were determined in each sample by measuring bacterial growth in cultures. The inhibition of bacterial growth was then measured by comparison with normal growth observed in microbes not treated with the testing samples.

### 2.8. Cell Cultures

The human skin cancer A375 cell line was purchased from the Bioresource Collection and Research Center (BCRC number: 60039, Hsinchu, Taiwan). This malignant melanoma A375 cell line was derived from a 54-year-old female. The cells were seeded in 96-well plates at a density of 7 × 10^3^ cells/well. The medium was then changed, and cells were maintained in either solvent alone (control cells) or in the presence of the indicated ginger extracts (50~1000 *μ*g/mL) in a final volume of 100 *μ*L within 10% FBS cellular culture medium. Each sample was added to a microplate and incubated under the same conditions as aforementioned.

### 2.9. Animal Material

In this study, the use of animals complied with the Guiding Principles in the Care and Use of Animals of the American Physiology Society and was approved by the National Kaohsiung Medical University and Use Committee. BALB/c nu/nu female mice (4-5 weeks) were purchased from BioLASCO Experimental Animal Center (Taiwan Co., Ltd). The mice were housed in Plexiglas cages in a temperature-controlled room (22 ± 1°C), on a 12-hour/12-hour light/dark schedule, and with free access to food and water. After one week, ten mice were randomly divided into 2 groups, control and drugs treatment groups. 

### 2.10. Xenograft Tumor Assay

The performance of xenograft tumor assay was described previously with minor modifications [24]. In brief, BALB/c nu/nu female mice were housed and the *in vivo *experiments were performed at the animal center (Kaohsiung Medical University, Kaohsiung, Taiwan). Mice were implanted subcutaneously with 1 × 10^7^ of A375 cells in 0.1 mL PBS injected subcutaneously in each mouse. Mice were treated four times a week with a subcutaneous injection of ginger extract (300 mg/kg) until sacrifice at day 35. The diameters of xenograft tumor were measured at 4 days intervals with vernier calipers and calculated as length × width^2^/2 in mm^3^ [25]. 

### 2.11. Blood Flow Measurement Using Laser Doppler Flowmetry

Mice used the tape fixed under test location and the moorLDI2 laser Doppler blood perfusion imagers (Moor Instruments Ltd, USA) was used to measure in the tumor periphery, and the digital color-coded images were analyzed to quantify blood flow in the back [26].

### 2.12. Statistical Analysis

The data were expressed as the mean value obtained in three experiments. Statistical comparisons were performed by Student *t*-test for paired values.

## 3. Results and Discussion

### 3.1. Determination of DPPH Radical Scavenging Capacity

The following purpose of this section was to survey the antioxidative properties of ginger extracts from supercritical fluid carbon dioxide extraction, and the testing samples were added at various concentrations (ranging from 100 *μ*g/mL to 5,000 *μ*g/mL). DPPH free radical scavenge testing mechanism is an acknowledged system by which antioxidants act to inhibit oxidation products; hence, this DPPH scavenging platform has been widely applied as one of the indicators for antioxidant abilities. In this DPPH assay procedure, antioxidants are able to decrease the stable purple DPPH radicals to the light yellow colored diphenyl-picrylhydrazine. The inhibition values of ginger extracts were listed in Table 1, and vitamin C at 100 *μ*M was as a positive control within this assay. It showed a dose-dependent manner that ginger extracts had moderate-high scavenging capability in DPPH assay. 

### 3.2. Metal Chelating Activity

The ferrous ion chelating activity of ginger extracts was described in Table 1. In ferrous ion chelating activity assay, ferrozine and Fe^2+^ can quantitatively form complexes and EDTA at 100 *μ*M applied as a positive control to possess ion chelating capacity of 95.4%. In the presence of chelating agents, the reagent complex formation is disrupted, resulting in a reduction in the dark red color of the complex. Ginger extracts at the dosage of 5,000 *μ*g/mL presented a middle-strong level on Fe^2+^ scavenging effects of 52.2%. 

### 3.3. Reducing Power

Ferric reducing antioxidant power assay is a simple and reliable platform, measuring the reducing potential of an antioxidant reacting with a ferric 2,4,6-tripyridyl-s-triazine Fe(III)-TPTZ complex, producing a dark blue colored ferrous Fe(II)-TPTZ complex by an adopted reductant. This complex has a conspicuous blue color which can be monitored at 700 nm. A higher absorbance in spectrogram indicates a higher ferric reducing power. In Table 1, ginger extracts demonstrated a minor ferric reducing power at the high dosage of 5,000 *μ*g/mL (0.35 ± 0.01), and the positive control was BHA at 100 *μ*M (1.92 ± 0.04). 

### 3.4. Antibacterial Effects of Extracts

The next experiment was to compare the antibacterial effects of ginger supercritical carbon dioxide fluid extracts.  Table 2 showed that ginger extracts were with efficient inhibition for the growth of *S. aureus*, *S. sobrinus,* and* E. coli*, but not *S. mutans*. The antibacterial assay illustrated that ginger extracts effectively inhibited both Gram-negative and Gram-positive bacteria. Generally, for each microbe, whether Gram-negative or Gram-positive, the growth phenomenon was time-dependently inhibited by the exposures of 5, 30, 60, 180, 300, and 900 seconds. 

### 3.5. The Melanoma Growth Inhibition on Xenograft Tumor by Ginger Extracts

**Figure 1:**
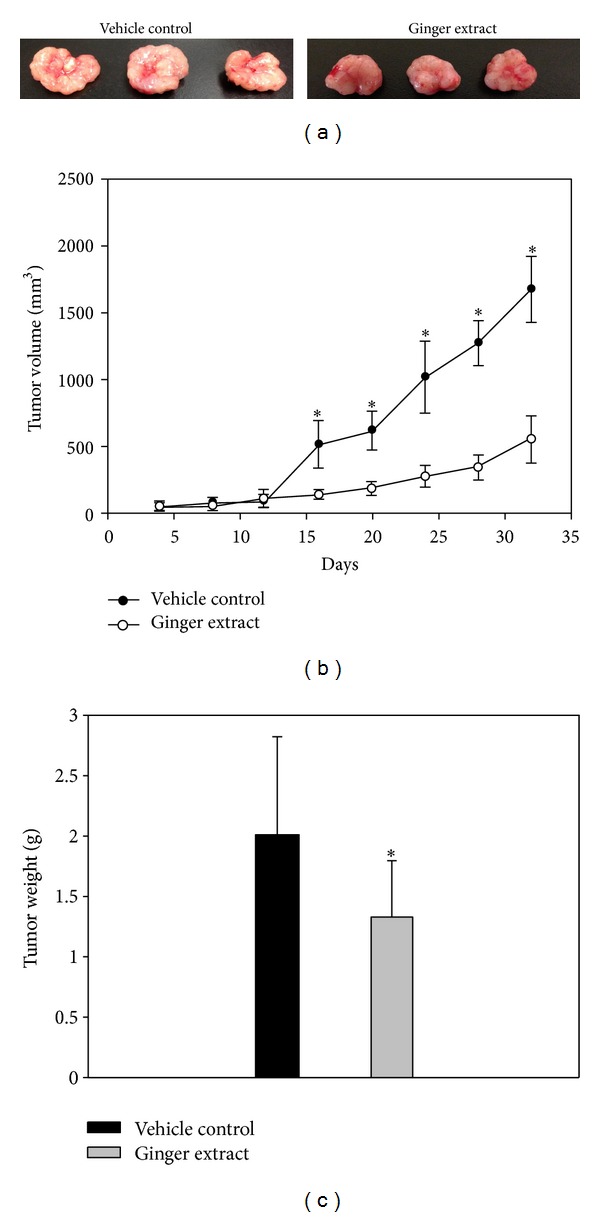
Ginger extract inhibits tumor growth in xenografts tumor assay. (a) Photo of mice and dissected tumors from vehicle control group and treated with ginger extracts 300 mg/kg. (b) Average tumor volume of vehicle control group versus ginger extracts and (c) average tumor volume weight were measured at the end of experiment. Five the number of samples were analyzed in each groups, and value represent the mean ± SD. Comparisons were subjected to Student's *t*-test. Significantly different at *P < 0.05.

The cellular proliferation assay illustrated antigrowth effects in nude mice on skin cancer cells (Figure 1). In this study, we examined the therapeutic efficacy of supercritical carbon dioxide fluid ginger extracts *in vivo* tumor xenograft by treating BALB/c nu/nu female mice bearing human malignant melanoma A375 cell lines, at a concentration of 300 mg/kg. After the establishment of palpable tumors (the mean tumor volume was around 50~100 mm^3^), animals received subcutaneous injections of ginger extracts four times for one week. After 35 days, we found out that the cellular A375 proliferation was reduced significantly compared to the vehicle control, and photographs in Figure 1(a) were the malignant cells of human skin cancer to show the inhibitory effects of ginger extracts. Obvious reducing properties were discovered at treatment conditions, and the quantification analysis of tumor volume and tumor weight was presented in Figures 1(b) and 1(c). We confirmed the inhibitory effect of ginger extracts on xenograft tumor assay, significantly. 

### 3.6. Effects of Ginger Extracts on the Mice Organ Weights and the Scanning Images of Laser Doppler Flowmetry *In Vivo*

**Figure 2:**
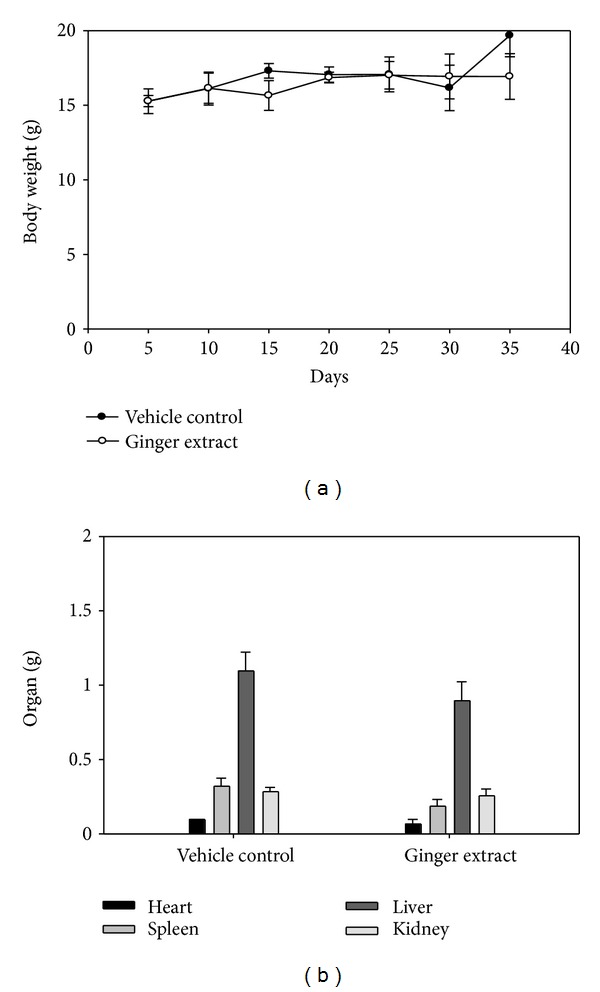
Effect of ginger extracts in organs in xenograft assay. (a) Average body weight of vehicle control group versus ginger extracts and (b) average organs (heart, spleen, liver, and kidney) of vehicle control group versus ginger extracts.

**Figure 3:**
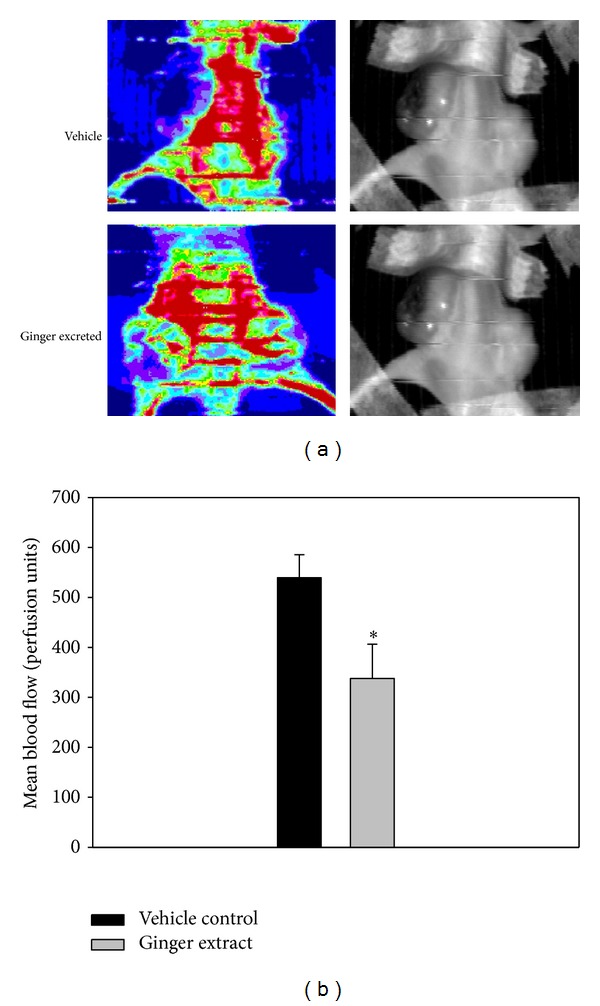
Effect of ginger extracts shows a laser Doppler scan of blood flow *in vivo.* (a) Photo of mice blood flow from vehicle control group and treated with ginger extracts 300 mg/kg. (b) Average blood flow of vehicle control group versus ginger extracts. Comparisons were subjected to Student's *t*-test. Significantly different at *P < 0.05.

Within mice receiving the treatment regimens, no gross signs of toxicity were observed (body weight, visible control inspection of general appearance, and microscopic examination of individual organs) (Figures 2(a) and 2(b)). In the tumors periphery, the blood flow was higher than that in the normal stomach tissues [27]. We analyzed the tumor blood flow around the experimental areas and showed that the blood flow of ginger extracts group was reduced compared to the vehicle control group (Figures 3(a) and 3(b)). Therefore, all of the results pointed out the promising potential of ginger extracts on antimelanoma treatment in the future.

## 4. Conclusion

Our research results showed that ginger extracts from supercritical fluid carbon dioxide extraction demonstrated DPPH free radical scavenging ability, reducing power and chelating property in dose-dependent manners. This research directly confirmed that the importance of ginger extracts had positively influencing levels of dietary antioxidants in the human body. Ginger extract proved the antibacterial activities against both gram-negative bacteria (*E. coli* and *S. sobrinus*) and gram-positive bacteria, (*S. aureus *and *S. mutans*), but *S. mutans* has no obvious effect in antibacterial activities. In animal experiments, the nude mice were pretreated with A375 melanoma cells, and the ginger extracts illustrated an obvious reduction in cell proliferations *in vivo* xenografts. To sum up, it was the first study of ginger extracts from supercritical fluid carbon dioxide to be potentially applied in food additive, infectious inhibition, or anticancer agents.

## Figures and Tables

**Table 1:** Antioxidant properties of ginger extracts on DPPH free radical scavenging, ferrous ion chelating, and reducing power ability assays.

Ginger (*μ*g/mL)	DPPH (%)	Reducing power (OD_700_ nm)	Chelating (%)
100	NS	NS	NS
250	4.5	NS	NS
500	18.1	12.7	NS
2500	31.9	21.5	0.17 ± 0.00
5000	66.0	52.2	0.35 ± 0.01

Vitamin C^a^	87.5	—	—
BHA^b^	—	95.4	—
EDTA^c^	—	—	1.92 ± 0.04

(—): no testing.

Vitamin C^a^ was used as a positive control on DPPH assay at 100 *μ*M.

EDTA^b^ was used as a positive control on metal chelating ability at 100 *μ*M. BHA^c^ was used as a positive control on reducing power at 100 *μ*M. Mean ± SD.

**Table 2:** Inhibition percentage of colony growth (CFU per milliliter) in *S. aureus*, *S. mutans*, *S. sobrinus,* and *E. coli. *

Ginger extract	Inhibition of bacterial growth (%)						
	Exposure time (seconds)						
	0	5	30	60	180	300	900
*S. aureus *	0	45.5 ± 5.9	58.5 ± 4.4	63.2 ± 9.2	64.1 ± 8.7	69.3 ± 10.2	74.8 ± 8.5
*S. mutans *	0	2.7 ± 1.5	9.3 ± 1.5	9.7 ± 3.2	14.4 ± 12.1	16.9 ± 4.3	19.6 ± 3.6
*S. sobrinus *	0	23.8 ± 9.2	34.6 ± 5.5	38.0 ± 5.5	43.2 ± 4.2	50.8 ± 6.7	56.2 ± 7.7
*E. coli *	0	15.6 ± 6.2	36.7 ± 4.0	45.4 ± 3.1	59.1 ± 2.9	75.7 ± 7.9	86.7 ± 7.7
